# Identification of Prefrontal Cortex and Amygdala Expressed Genes Associated With Sevoflurane Anesthesia on Non-human Primate

**DOI:** 10.3389/fnint.2022.857349

**Published:** 2022-07-01

**Authors:** Yanyong Cheng, Siyu Liu, Lei Zhang, Hong Jiang

**Affiliations:** Department of Anesthesiology, Shanghai Ninth People’s Hospital, Shanghai Jiao Tong University School of Medicine, Shanghai, China

**Keywords:** sevoflurane, primate, prefrontal cortex, amygdala, RNA sequencing

## Abstract

Clinical trials and animal studies have indicated that long-term use or multiple administrations of anesthesia may lead to fine motor impairment in the developing brain. Most studies on anesthesia-induced neurotoxicity have focused on the hippocampus and prefrontal cortex (PFC); however, the role of other vital encephalic regions, such as the amygdala, is still unclear. Herein, we focused on sevoflurane, the most commonly used volatile anesthetic in infants, and performed a transcriptional analysis of the PFC and amygdala of macaques after multiple exposures to the anesthetic by RNA sequencing. The overall, overlapping, and encephalic region-specific transcriptional patterns were separately analyzed to reveal their functions and differentially expressed gene sets that were influenced by sevoflurane. Specifically, functional, protein–protein interaction, neighbor gene network, and gene set enrichment analyses were performed. Further, we built the basic molecular feature of the amygdala by comparing it to the PFC. In comparison with the amygdala’s changing pattern following sevoflurane exposure, functional annotations of the PFC were more enriched in glial cell-related biological functions than in neuron and synapsis development. Taken together, transcriptional studies and bioinformatics analyses allow for an improved understanding of the primate PFC and amygdala.

## Introduction

The safety of anesthetic exposure in the developing brain of children and infants has attracted significant public attention. Increasing clinical trials have shown that the administration of general anesthesia is associated with deficits in long-term motor coordination and social behavior in children and infants (Ing and Brambrink, 2019; Zaccariello et al., 2019; Feng et al., 2020; Ing et al., 2020; Kobayashi et al., 2020; Walkden et al., 2020). Sevoflurane, the most common anesthetic used in children, is the main object of interest in the study of neurotoxicity of general anesthetics.

Sevoflurane, a volatile anesthetic agent, can disrupt consciousness and cognition. It has also been shown to lead to synaptic and myelin damage, neuroinflammation, excitatory neuron abnormality, and neuronal apoptosis in the brains of young animals (Xie et al., 2020; Zhao et al., 2020). To explore the molecular mechanism underlying anesthesia and sevoflurane-induced deficiencies, a number of transcriptome studies using RNA sequencing (RNA-seq) techniques have been conducted.

Various brain regions were included in such studies, especially the striatum, hypothalamus, prefrontal cortex (PFC), and hippocampus (Song et al., 2019; Zhang et al., 2019a; Xie et al., 2020; Yamamoto et al., 2020; Zhao et al., 2020; Zhang L. et al., 2021). The differentially expressed genes (DEGs) identified by transcriptome studies and verified by subsequent experiments have helped to explain the observed adverse effects of sevoflurane.

The PFC has extensive reciprocal connections with wake-promoting centers in the brainstem and diencephalon (Briand et al., 2007; Pal et al., 2018); indeed, cholinergic stimulation of the PFC can restore wake-like behavior following sevoflurane anesthesia (Pal et al., 2018). Sevoflurane exposure has been shown to induce behavioral deficits, poor memory performance, and neurofunctional abnormalities by disrupting excitatory neurons in the PFC (Xu et al., 2018; Xie et al., 2020; Zhao et al., 2020). Our previous study found that sevoflurane-induced impairment in m6A-mediated mRNA translation in the PFC was related to fine motor deficits (Zhang L. et al., 2021). Sevoflurane exposure contributed to higher tau concentration, lower brain mitochondrial metabolism, aberrant non-coding RNA expression, and hypermethylation in the hippocampus, which result in impairment of neurocognition, neurogenesis, learning, and memory (Hu et al., 2019; Shao and Xia, 2019; Yu et al., 2020; Fan et al., 2021); indeed, the hippocampus is heavily involved in learning, cognition, memory acquisition, and storage (Shao and Xia, 2019). The amygdala is also involved in endocrine modulation and autonomic nervous activity. However, an abnormal gene expression in the amygdala after sevoflurane exposure has not been thoroughly studied.

The amygdala is a brain structure not only involved in emotional responses, but also in social and parenting behaviors (Gilpin et al., 2015; Janak and Tye, 2015; Chen et al., 2019). Research on memory modulation has shown that subjective sentiment is likely to affect the strength of our memories (Roozendaal and McGaugh, 2011); recent findings have shown that the amygdala plays an important role in memory, especially in emotionally significant experiences (Roozendaal et al., 2009). The formation of emotional memories depends on dynamic amygdala-hippocampus interactions (Richter-Levin and Akirav, 2000). Hassa et al. (2017) suggested a novel mechanistic direct link between dysregulated emotional processing and the motor control circuitry in conversion disorders. In addition to emotional control, such studies revealed the multifunctionality of the amygdala, such as motor control ability. Furthermore, it is worth noting the following behavioral contributions of the amygdala-PFC network: learned methods and avoidance, foraging, predator defense, social signaling, and social decision-making (Yizhar and Klavir, 2018; Gangopadhyay et al., 2021; Murray and Fellows, 2022).

In this study, a primate model with multiple sevoflurane exposures which used in our previous researches (Zhang et al., 2019a; Cheng et al., 2020; Zhang L. et al., 2021; Chen et al., 2022) to study the mechanism of sevoflurane-induced neurotoxicity was applied. We explored the gene expression profiling in the amygdala and PFC of rhesus macaques (*Macaca mulatta*), with or without sevoflurane exposure, by RNA-seq to elucidate the previously identified dysregulated genes and pathways related to sevoflurane exposure. Intriguingly, the amygdala and PFC have been shown to include different DEGs after sevoflurane exposure. The intersection of the DEGs set might uncover the broad mechanism of sevoflurane exposure. Moreover, discovering an even greater number of dysregulated genes, especially those found in the amygdala, might further elucidate the specific functions of the amygdala.

## Materials and Methods

### Collection of Amygdalae and Prefrontal Cortexs From Macaques

The animal studies were performed according to the guidelines and regulations of the Institute of Laboratory Animal Science, Peking Union Medical College and Chinese Academy of Medical Sciences (PUMC & CAMS, Beijing, China). The macaques were purchased from PUMC & CAMS. Efforts were made to minimize the number of macaques. The use of macaques in research at the Institute of Laboratory Animal Science was approved by the Institutional Animal Care and Use Committee (protocol number #XC17001). Samples of PFC were from 4 female macaques [2 females in the control (Ctrl) group, 2 females in the sevoflurane (SEV) group]. For amygdala samples, one female and one male macaque in the Ctrl group and one female and one male macaque in the SEV group. Only the macaques in the experimental group received sevoflurane anesthesia, as described in our previous studies (Cheng et al., 2020; Zhang L. et al., 2021; Chen et al., 2022). Briefly, the macaques received 6–8% anesthetic sevoflurane with 100% oxygen for the induction (2–4 min) of general anesthesia and then received 2.5–3% sevoflurane and 100% oxygen with endotracheal intubation for 4 h to maintain general anesthesia on postnatal day (P) P7 and then on P21 and P35, respectively. At the end of the last 4-h sevoflurane anesthesia session (P35), the macaques were euthanized by perfusion and decapitation. The corporal temperatures of the rhesus macaques were maintained by placing them in a warm box (37^°^C). The peripheral capillary oxygen saturation and heart rate were monitored. The macaques in the Ctrl group received three maternal separations of a duration equal to that in the SEV group (4 h) and underwent the same sampling procedure as previously described. The macaques in control condition received 3% sevoflurane briefly (<5 min) before culling for harvesting the amygdala and PFC at the end of the third round of sevoflurane anesthesia on P35. The left side of ventromedial PFC was mainly sampled for RNA-seq. Only the left central portion of the amygdala was sampled. All collected samples were quickly placed in freezing tubes for storage at liquid nitrogen temperature.

### RNA Sequencing

Total RNA was extracted by Trizol reagent (Invitrogen, United States) separately. The RNA quality was checked by Agilent 2200 and kept at -80^°^C. The cDNA libraries were constructed for each RNA sample using the TruSeq Stranded mRNA Library Prep Kit (Illumina Inc., United States) according to the manufacturer’s instructions. Generally, the protocol consists of the following steps: Poly-A containing mRNA was purified from 1 μg total RNA using oligo dT magnetic beads and fragmented into 200–500 bp using divalent cations at 94^°^C for 5 min. The cleaved RNA fragments were used for first- and second-strand complementary DNA (cDNA) synthesis. dUTP mix was used for second-strand cDNA synthesis, which allows for the removal of the second strand. The cDNA fragments were end repaired, A-tailed and ligated with indexed adapters. The ligated cDNA products were purified and treated with uracil DNA glycosylase to remove the second-strand cDNA. Purified first-strand cDNA was enriched by PCR to create the cDNA libraries. Target bands were harvested through 2% agarose gel electrophoresis and quantified by Agilent2200. Then the cDNA libraries were used for 150 bp paired-end sequencing on Illumina HiSeq XTen platform.

After removing the adaptor sequences and low-quality reads, clean reads were obtained from the raw reads. The clean reads were then aligned to Rhesus monkey genome (Mmul_10 Ensembl98, Ensembl) using the Hisat2 (Kim et al., 2015). HTseq was used to get gene Counts and FPKM method was used to determine the gene expression (Anders et al., 2015). EB-Seq algorithm was applied to filter the DEGs, after the significant analysis, *P*-value and FDR analysis under the following criteria: (i) Fold Change > 2 or <0.5; (ii) *P*-value < 0.05, FDR < 0.05. Special criteria were listed separately.

During the analysis, the number of DEGs in the amygdala of macaques that have been exposed to sevoflurane was downsized to 898 based on more strict criteria: (i) Fold Change > 10 or <0.1; (ii) FDR < 0.01 (the working flow was shown in Supplementary Figure 1).

### Quantitative Real-Time PCR

The amygdalae and PFCs of macaques were collected as described above-mentioned. Total RNA was harvested by Trizol (Invitrogen, United States) and reversed into cDNA by Prime-Script RT reagent Kit (Takara, Japan) according to their manufacturer’s instructions. The relative expressions of mRNA were detected by Standard SYBR-Green method on the Real-Time PCR System (Applied Biosystems ABI7500, United States), normalized to the level of GAPDH. The results were presented as 2^–ΔΔCt^. The primer sequences were listed as follow: DCC: F: GCCGACCCTAGAAAGTGC, R: TTCCTGCTCCGAAACCTC; PLCB1: F: TTGGCTGCTTTGACACTG, R: CTGCTGGAGTTG TTCTCACT; HOMER1: F: TAGTAGCCAAGCAAACGC, R: GT TGTCCCTCCAGGTCTT; NF1: F: GCCTTCCGTTCCAGTT AC, R: CATGCCTCCATGATCTCC; LRP6: F: AAGAACCAG CACCACAGG, R: ACCAAGAGGCACAGAAGC; and PPP3R1: F: GATGGGAATGGAGAAGTA, R: CATAGATACGGAAAG CAA.

### Functional Annotation

Metascape (Zhou et al., 2019) provides a common resource for analyzing system-level datasets. In this study, it was used to further validate the enrichment of DEGs and transcription factors (TFs) of macaques after multiple sevoflurane anesthesia. In Metascape analysis, significant terms were hierarchically clustered into a network tree based on Kappa-statistical similarities among their gene memberships. Each term is represented by a circle node, where its size is proportional to the number of input genes fall into that term, and its color represent its cluster identity. Terms with a kappa similarity score > 0.3 are linked by an edge.

### Gene Ontology Enrichment Analysis and Pathway Analysis

Those identified DEGs were subjected to Gene ontology (GO) enrichment analysis and Pathway enrichment analyses as described. GO and functional analysis using the GO annotations from NCBI, DAVID (Huang da et al., 2009a,b) and the Gene Ontology. Fisher’s exact test was applied to identify the significant GO categories (*P*-value < 0.05). According to Kyoto encyclopedia of genes and genomes (KEGG) database, we used the Fisher’s exact test to select the significant pathway, and the threshold of significance was defined by *P*-value < 0.05.

### Protein–Protein Interaction Network

The Search Tool for the Retrieval of Interacting Genes/Proteins (STRING) v11 (Szklarczyk et al., 2019) was used to construct the protein–protein interaction (PPI) network. After multiple administrations of sevoflurane anesthesia, PPI network analysis was performed using STRING for DEGs and TFs in the macaques’ amygdalae and PFCs to explore their interactions. In order to obtain objective and reliable results, we restricted the selection of DEGs and TFs to high-throughput lab experiments and previous knowledge found in databases. In addition, the minimum interaction score was set at a high confidence level (0.9).

### Gene Set Enrichment Analysis

For the identification of enriched DEG signatures, we used the gene set enrichment analysis (GSEA) tool (v4.1.0) from the Broad Institute at the Massachusetts Institute of Technology (Mootha et al., 2003; Subramanian et al., 2005). This tool derives its power by focusing on gene sets that share common biological functions or regulation, revealing several shared biological pathways. The analysis was performed by comparing the DEGs obtained from the PFC and amygdala between the SEV and Ctrl groups.

### Functional Analysis and Genes Enrichment Analysis

To identify the functions of the aforementioned DEGs and their enrichment in these functions, bioinformatics analysis was performed by Cytoscape ClueGO bioinformatics tool (v2.5.8; Bindea et al., 2009). Regarding the statistical approach of the enrichment analyses by Cytoscape, a *P*-value < 0.05 and kappa coefficient of 0.4 were considered as threshold values and correction was performed by Bonferroni test.

### Analysis of Differentially Expressed Transcription Factors

The prediction of the binding reaction between the transcription factors (TFs) and their target genes that were changed significantly after sevoflurane anesthesia was performed by Cytoscape iRegulon bioinformatics plugin (Janky et al., 2014).

## Results

### Different Gene Expression Patterns in Macaques’ Amygdalae and Prefrontal Cortexs After Sevoflurane Anesthesia

**FIGURE 1:**
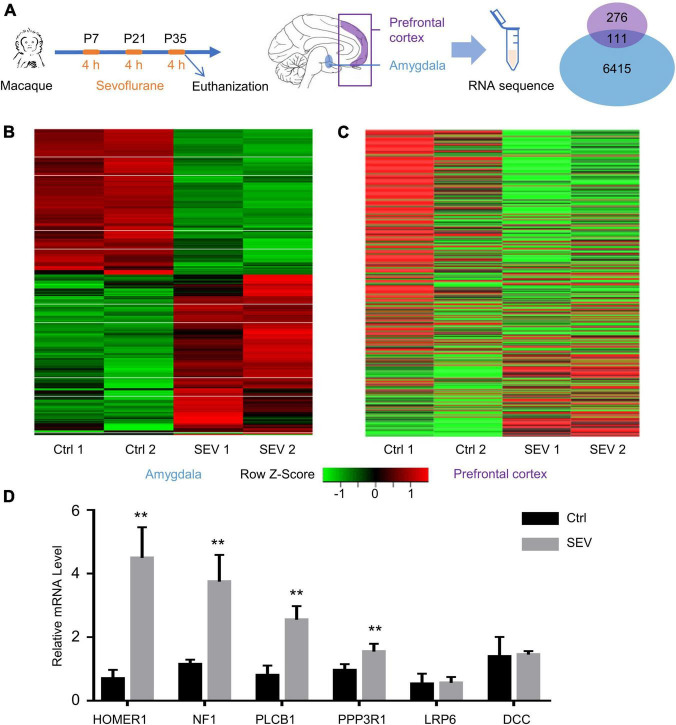
Differentially expressed genes (DEGs) in macaques’ amygdala and prefrontal cortex (PFC) after multiple sevoflurane anesthesia. **(A)** Experimental design. The rhesus macaques received 2.5–3% sevoflurane and 100% oxygen for 4 h on postnatal day (P) 7 and then on P21 and P35, respectively. The amygdala and PFC were harvested at the end of the third round of sevoflurane anesthesia on P35. Venn Diagram representation of 387 and 6,526 DEGs were identified from amygdala and PFC after multiple sevoflurane anesthesia, respectively. One hundred and eleven genes were overlapped. **(B,C)** Heatmap of significant DGEs in amygdala and PFC. **(D)** The levels of relative mRNA were assessed via qPCR as described. HOMER1, NF1, PLCB1, PPP3R1 mRNA expression increased in the macaques’ amygdala after multiple sevoflurane exposure (*n* = 5, *p* < 0.01, one-way ANOVA). Relative mRNA level of LRP6, DCC didn’t alter after sevoflurane exposure (*n* = 5, *p* = 0.7127 and *p* = 0.7065, respectively, one-way ANOVA). (** *p* < 0.01).

RNA sequencing analysis was performed on samples from the Ctrl and SEV groups to identify any differences in the gene expression patterns in the macaques’ amygdala and PFC after multiple administrations of sevoflurane anesthesia, including two of 4-h exposures on P7 and P21 and an acute 4-h exposure immediately before harvesting PFC and amygdala on P35. A total of 387 and 6526 DEGs between the SEV and Ctrl groups were identified in the amygdala and PFC of macaques, respectively, (Figure 1A). The heat map of the sequencing data clearly separated the Ctrl and SEV groups (Figures 1B,C). Further, parts of the obtained DEGs were verified by PCR (Figure 1D).

**FIGURE 2:**
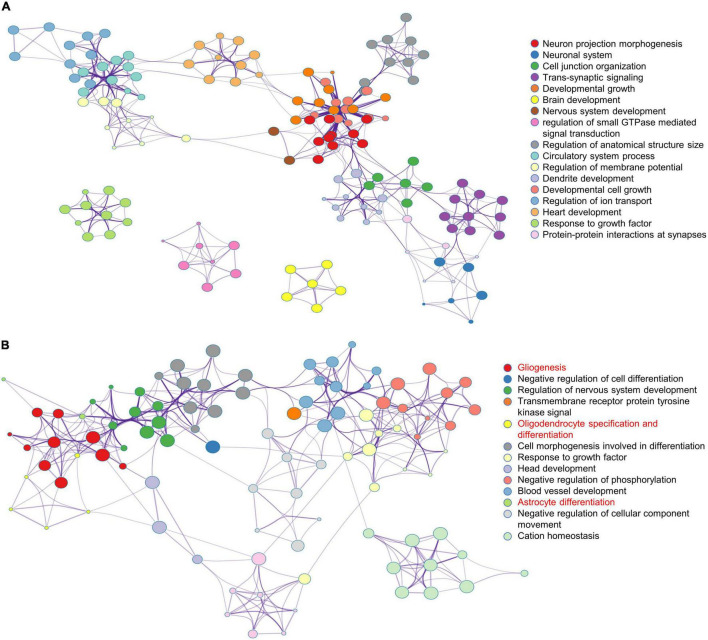
Functional annotation of DEGs in macaques’ amygdala and PFC after multiple sevoflurane anesthesia using Metascape. **(A)** The network plot of 898 DEGs in macaques’ amygdala after multiple sevoflurane anesthesia. **(B)** The network plot of 387 DEGs in macaques’ PFC after multiple sevoflurane anesthesia.

To further analyze the effects of multiple administrations of sevoflurane anesthesia on the amygdala and PFC of macaques, functional annotation of DEGs was performed using Metascape. Compared to the respective gene sites in the Ctrl group, the gene functional terms of 898 DEGs in the amygdala of macaques in the SEV group were mainly enriched in the regulation of neuron projection morphogenesis and brain, nervous system, and dendrite development (Figure 2A). Further, the 387 DEGs in the PFC of macaques in the SEV group were primarily enriched in gliogenesis, oligodendrocyte specification and differentiation, and astrocyte differentiation (Figure 2B).

Gene ontology and KEGG analyses were used to confirm the enrichment of functions of the 898 DEGs in the amygdala after sevoflurane anesthesia (Supplementary Figures 2, 3). To further understand and verify their function, GSEA analysis revealed that DEGs in the amygdala and PFC of macaques in the SEV group were more enriched in gene concentrations of nervous system related functions (axonogenesis, neuron differentiation, and neuron development) than in those of macaques in the Ctrl group (Supplementary Figure 4). Specifically, gene sets associated with central nervous system neuron axonogenesis, neuron axonogenesis neuron differention, and neuron development. Gene sets associated with major types of astrocytes and fetal cerebrum astrocytes were differentially enriched in DEGs of the macaque amygdala and PFC after multiple administrations of sevoflurane anesthesia (Supplementary Figure 4). PPI networks were established separately by 898 DEGs in the amygdala (Supplementary Figure 5A) and 387 DEGs in the PFC (Supplementary Figure 5B) of macaques in the SEV group to describe the potential interactions. Hub genes selected by Cytoscape are marked in red.

### Bioinformatics Analysis of the Overlapped Differentially Expressed Genes Between the Macaque Prefrontal Cortex and Amygdala After Multiple Sevoflurane Anesthesia

**FIGURE 3:**
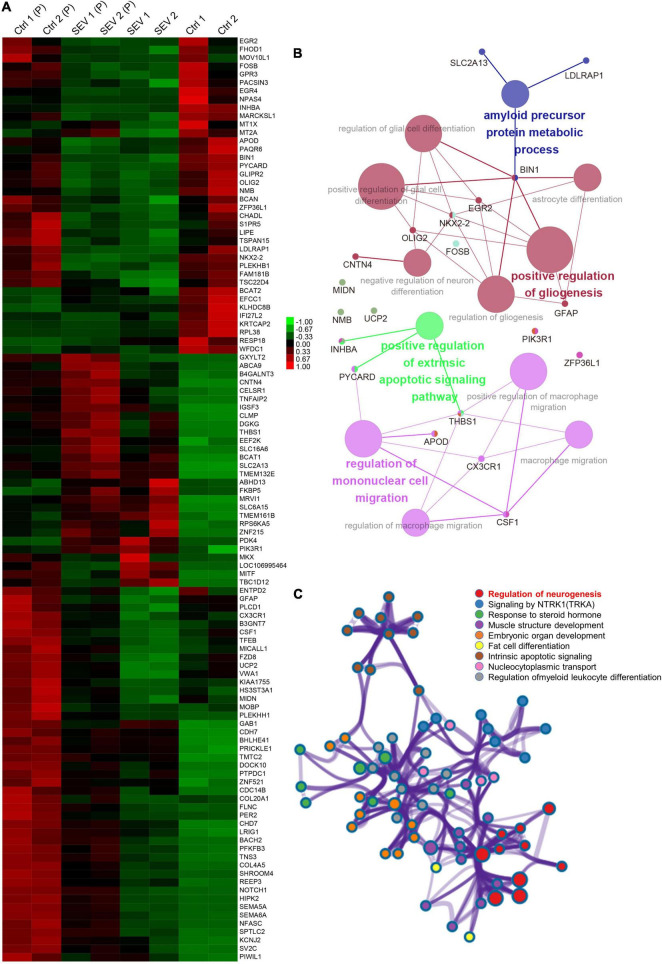
The overlapped 111 genes differently express in PFC and amygdala after multiple sevoflurane anesthesia. **(A)** Heatmap of the overlapped 111 genes. Samples in amygdala group, respectively, named Ctrl1, Ctrl2 and SEV1, SEV2. Samples in PFC group, respectively, named Ctrl1(P), Ctrl2(P) and SEV1(P), SEV2(P). **(B)** Significant enriched gene ontology (GO) and Kyoto encyclopedia of genes and genomes (KEGG) pathway terms of the overlapped DEGs in macaques’ amygdala and PFC after multiple sevoflurane anesthesia. **(C)** Functional annotation of transcription factors (TFs) in the overlapped 111 genes after multiple sevoflurane anesthesia using Metascape.

After multiple administrations of sevoflurane anesthesia, 111 DEGs appeared simultaneously in the PFC and amygdala of macaques (Figure 1A). The transcriptional patterns of these 111 DEGs were analyzed using a heatmap to infer the common function of two different regions (Figure 3A). The GO and KEGG network diagrams (Figure 3B) and bar plots (Supplementary Figure 6) display the results of the functional enrichment analysis to better understand the underlying biological functions and pathways associated with overlapping DEGs. To further predict the biological processes in which these 111 DEGs may be involved, functional annotation was performed using Metascape. The network showed that the TFs of these overlapping DEGs were mainly enriched in the regulation of neurogenesis (Figure 3C). PPI networks of 111 DEGs (Supplementary Figure 7A) and TFs (Supplementary Figure 7B) were constructed to gain insight into possible cellular mechanisms.

### Primate Prefrontal Cortex-Special Functional Analysis of Differentially Expressed Genes After Multiple Sevoflurane Anesthesia

**FIGURE 4:**
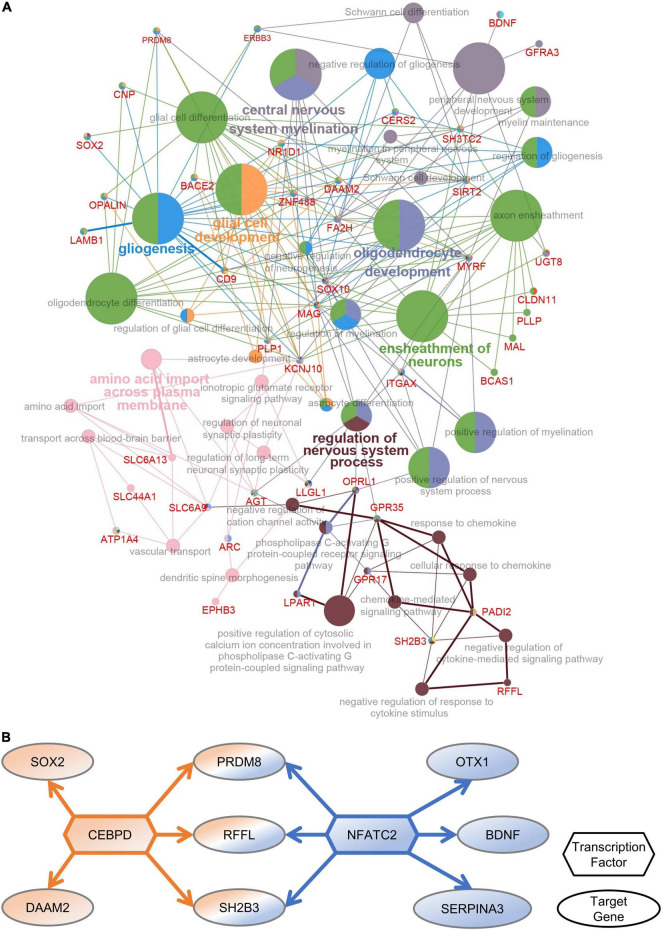
PFC-special function analysis. **(A)** The network of DEGs and related GO classes. **(B)** The network of PFC-specific DEGs and differentially expressed TFs.

To identify the functions of DEGs in the PFC after multiple rounds of sevoflurane anesthesia and their enrichment in these functions, 276 DEGs were analyzed using Cytoscape. The results revealed that GO classes were enriched in biological processes such as central nervous system myelination, oligodendrocyte and glial cell development, gliogenesis, ensheathment of neurons, and regulation of nervous system processes (Figure 4A). The network of PFC-specific DEGs and differentially expressed TFs showed possible regulation between them (Figure 4B). CEBPD and NFATC2 were shown to play a central role in sevoflurane-induced dysfunction in the primate PFC. Functional annotation was also performed using Metascape; the differentially expressed TFs were enriched in glial cell development and the Wnt signaling pathway (Supplementary Figure 8).

### Primate Amygdala-Special Functional Analysis of Differentially Expressed Genes After Multiple Sevoflurane Anesthesia

**FIGURE 5:**
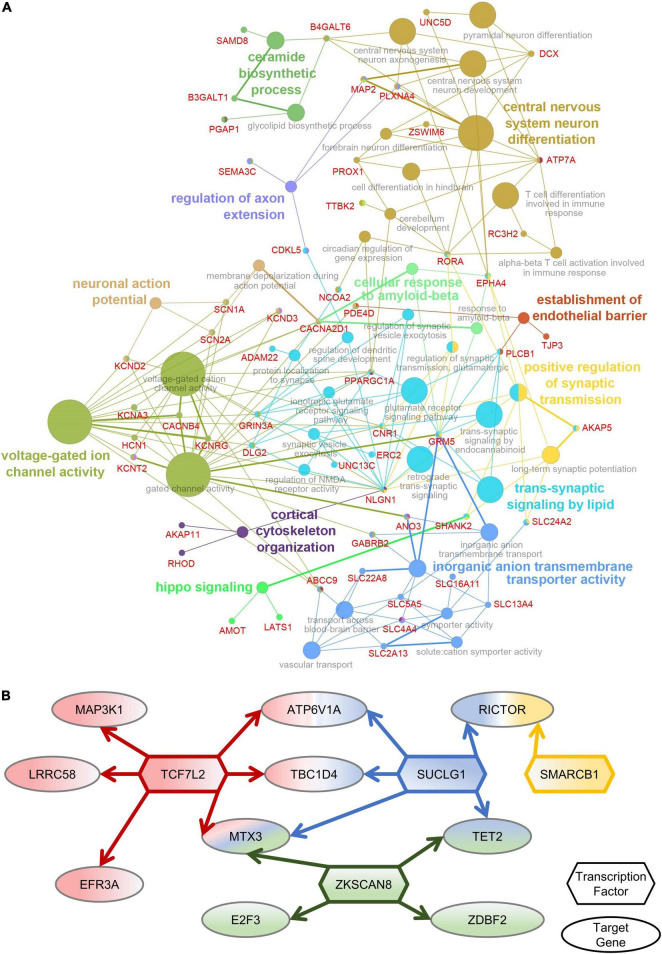
Amygdala-special function analysis. **(A)** The network of DEGs and related GO classes. **(B)** The network of Amygdala-specific DEGs and differentially expressed TFs.

To determine the functions of amygdala-specific DEGs in macaques after multiple rounds of sevoflurane anesthesia and their enrichment, 787 DEGs were analyzed using Cytoscape. The interaction network described the enrichment of biological process annotations, such as central nervous system neuron differentiation, ceramide biosynthetic process, voltage-gated ion channel activity, and *trans*-synaptic signaling by lipids (Figure 5A). The results of amygdala-specific DEGs and differentially expressed TFs indicated a connection between TFs and target genes (Figure 5B). TFs, such as TCF7L2, SUCLG1, ZKSCAN8, and SMARCB1, were predicted to play a pivotal role in sevoflurane-induced dysfunction in the primate amygdala. Functional annotation conducted using Metascape supplements this analysis (Supplementary Figure 9).

## Discussion

In this study, a set of sevoflurane-related regulated genes in the primate amygdala was obtained from RNA-Seq data. The GO enrichment and KEGG pathway analyses of this gene set revealed a correlation between sevoflurane administration and regulation of the nervous system. Region-dependent functions were the focus of this study. Sevoflurane-related gene regulation in the amygdala was found to be 20 times greater than that in the PFC. PPI networks describe the potential interactions. Further, GSEA analysis revealed that DEGs were enriched in the gene concentrations of related functions. The results of region-specific DEGs and differentially expressed TFs indicated a connection between TFs and target genes; the analyzed overlaps exhibited the same changing pattern. The above results illustrated the difference in DEG function annotation of the macaque amygdala and PFC after multiple administrations of sevoflurane anesthesia. Besides the functional terms of neuron development, DEGs in the PFC focused on glial cell-related biological functions. Altogether, the findings suggest that the amygdala plays a unique role in the sevoflurane-induced influence of the nervous system.

The growing stage of the human nervous system is from the third trimester of pregnancy to 2–3 years after birth. Environmental stimulation at the infant stage may affect the structure of the developing brain, as well as its function. Therefore, it is important to investigate the side effects and mechanisms of general anesthetics on the development of the nervous system in infants and young children. A few clinical studies have focused on this, with the secondary analysis of the Mayo Anesthesia Safety in Kids (MASK) being one of the most relevant. The MASK study indicated that multiple, but not single, inductions of general anesthesia before the age of three are associated with a specific pattern of deficits (processing speed, fine motor ability, motor coordination, and visual-motor integration) in neuropsychological tests, instead of general intellectual damage (Ing and Brambrink, 2019; Zaccariello et al., 2019).

A population-based birth cohort study, comprising 13,433 children, revealed that manual dexterity performance and social communication scores were lower in children with multiple exposures to anesthesia than in those without (Walkden et al., 2020). A Japanese birth cohort study suggested that the risk of delays in all five developmental domains (communication, gross motor, fine motor, problem solving, and personal-social) was significantly increased (three times or more) in infants who underwent surgery under general anesthesia than in those who did not (Kobayashi et al., 2020). In addition, children under 5 years of age were found to be 37% more likely to require subsequent persistent use of medications for attention deficit and hyperactivity disorder than unexposed children (Ing et al., 2020). Further, the results of a Chinese population-based cohort study suggested that children exposed to general anesthesia before 2 years of age have an increased risk of developmental delay than unexposed children (Feng et al., 2020).

Moreover, clinical trials have also produced adverse results. The results of the Pediatric Anesthesia Neurodevelopment Assessment study found that there was no significant difference in the intelligence quotient (IQ) and cognitive functions between children with or without exposure to general anesthesia (Sun et al., 2012). However, the results of the General Anesthesia compared to Spinal anesthesia trial showed that, upon undergoing a single short operation, infants and children less than 60 weeks of age present no difference in their IQ and neurodevelopment after 2–5 years, whereas those who did not receive general anesthesia presented improvements (Davidson et al., 2016; McCann et al., 2019). Relevantly, all trials with negative results focused on single-exposure and short-time anesthesia; however, multiple sevoflurane exposures can specifically cause injuries.

In the present study, we discovered a difference in sevoflurane-related effects between the amygdala and PFC of macaques. In the PFC, 276 genes were found to be regulated, whereas 6415 DEGs were found in the amygdala. Currently, it is unclear why there was about a 20-fold difference in the quantity of DEGs between the two brain regions. However, the large number of amygdala-specific dysregulation genes is more likely to reveal the changes in neuron function. Further, 111 overlapped genes were identified. Despite the different basic expression quantities of these genes, the DEGs in both brain regions had the same expression pattern. These findings confirm that sevoflurane affects gene expression at the transcriptome level in multiple brain regions. Therefore, our study is very useful to deepen our understanding of sevoflurane-induced influence on different brain regions.

Among the TFs corresponding to 276 different genes in the PFC, the brain-derived neurotrophic factor (BDNF) plays a key role in neurodevelopmental disorders and behavioral changes caused by general anesthesia. Reduced neuronal proliferation and circuit damage have been found to be associated with decreased levels of BDNF in the brain under general anesthesia (Obradovic et al., 2018; Zhao et al., 2018). Overexpression of BDNF or stimulation of astrocytes to release BDNF can reduce neurodevelopmental toxicity induced by general anesthesia (Liu et al., 2017; Wang et al., 2018; Zhang et al., 2019b). Indeed, general anesthetics reduce the level of BDNF in the developing brain, which may be related to epigenetic modifications (Dalla Massara et al., 2016; Wu et al., 2016). Interestingly, BDNF also plays a role in postoperative delirium and postoperative cognitive dysfunction in elderly mice (Lu et al., 2020; Qiu et al., 2020). Altogether, these findings may suggest a common mechanism by which general anesthesia leads to brain injury.

Ten-eleven translocation 2 (TET2), a TF targeted by the amygdala’s differential gene, which mediates methylated CG demethylation during Mammalian brain development (Lister et al., 2013), is worth further investigation. In addition, 5-hydroxymethylcytosine (5hmC) is emerging as an active contributor to demethylation modification (Guo et al., 2011a,b). TET proteins, the dioxygenase for DNA hydroxymethylation, can produce 5hmC from 5-methylcytosine (Iyer et al., 2009; Tahiliani et al., 2009). TET1-mediated DNA hydroxymethylation is required for myelination and remyelination in the mouse brain (Zhang M. et al., 2021). TET-mediated active DNA demethylation plays vital roles in various biological processes, not only in development, but also in diseases. Given that recent studies have linked TET mutations to neurodegenerative disease (Cochran et al., 2020; Marshall et al., 2020), it is evident that further exploration of TET-mediated DNA demethylation will continue to enhance our understanding of the relationship between general anesthesia and the brain.

A primate model with multiple sevoflurane exposures was used to illustrate the anesthesia-induced influence on PFC and amygdala. It is remarkably that this model contained both acute and chronic sevoflurane exposures as the sampling time was immediately at the end of the last sevoflurane anesthesia session. If we want to focus on the effect of multiple chronic sevoflurane exposures, the sampling time should be appropriately extended. As in previous studies, results obtained from macaques are closer to those of humans. However, because of animal welfare and associated costs, macaques cannot be used to explore the mechanisms of interest in basic experiments. On the other hand, rodent experiments involve the dilemma of not being transferrable to the clinic. Therefore, we included as few rhesus macaques as possible to obtain a regulated gene set that was closest to that of humans through high-throughput sequencing. Because of the difficulty in collecting samples from macaques, we did not acquire a large sample size, which is a limitation to our statistical analysis. Additionally, sex differences were not considered in this study although recent studies have proposed different sevoflurane-related susceptibilities of different sexes (Ju et al., 2019; Cabrera et al., 2020; Chastain-Potts et al., 2020). The screening of target genes and the exploration of sevoflurane-related function in amygdala would be our directions of further research.

To conclude, this study on gene expression regulation was performed on the amygdala and PFC of primate brains using RNA-seq. The transcriptome changes in the specimens of macaques that were administered sevoflurane provided evidence in support of the relevant role of the amygdala in the biological processes influenced by sevoflurane. These animal-derived results might improve our understanding of neuronal injury caused by sevoflurane.

## Data Availability Statement

The datasets analyzed for this study can be found in the Gene Expression Omnibus (GSE119625 and GSE192783); (GSE192783 currently private).

## Ethics Statement

This animal study was reviewed and approved by the Institutional Animal Care and Use Committee (protocol number #XC17001).

## Author Contributions

LZ and HJ conceived the idea and designed the research. YC and SL performed the research and drafted the manuscript. YC analyzed the data. All authors approved the final manuscript.

## Conflict of Interest

The authors declare that the research was conducted in the absence of any commercial or financial relationships that could be construed as a potential conflict of interest.

## Publisher’s Note

All claims expressed in this article are solely those of the authors and do not necessarily represent those of their affiliated organizations, or those of the publisher, the editors and the reviewers. Any product that may be evaluated in this article, or claim that may be made by its manufacturer, is not guaranteed or endorsed by the publisher.
