# Supplementary material for: Identification of Prefrontal Cortex and Amygdala Expressed Genes Associated With Sevoflurane Anesthesia on Non-human Primate
Source: Front Integr Neurosci. 2022 Jul 1;16:857349. doi: 10.3389/fnint.2022.857349 (PMC9286018; doi:10.3389/fnint.2022.857349)
Supplement: Supplementary file 1 [file Data_Sheet_1.docx]

Identification of prefrontal cortex and amygdala expressed genes associated with sevoflurane anesthesia on non-human primate

Yanyong Cheng^1^, Siyu Liu^1^, Lei Zhang^1*^, Hong Jiang^1*^

^1^Department of Anesthesiology, Shanghai Ninth People’s Hospital, Shanghai Jiao Tong University School of Medicine, Shanghai 200011, China

* Correspondence:
Corresponding Author
Lei Zhang, weiymzhl@126.com, Hong Jiang, [jianghongjiuyuan@163.com](mailto:jianghongjiuyuan@163.com).

Yanyong Cheng and Siyu Liu contributed equally to this manuscript.

Keywords: sevoflurane, primate, prefrontal cortex, amygdala, RNA sequencing.

SUPPLEMENTARY FIGURES

**
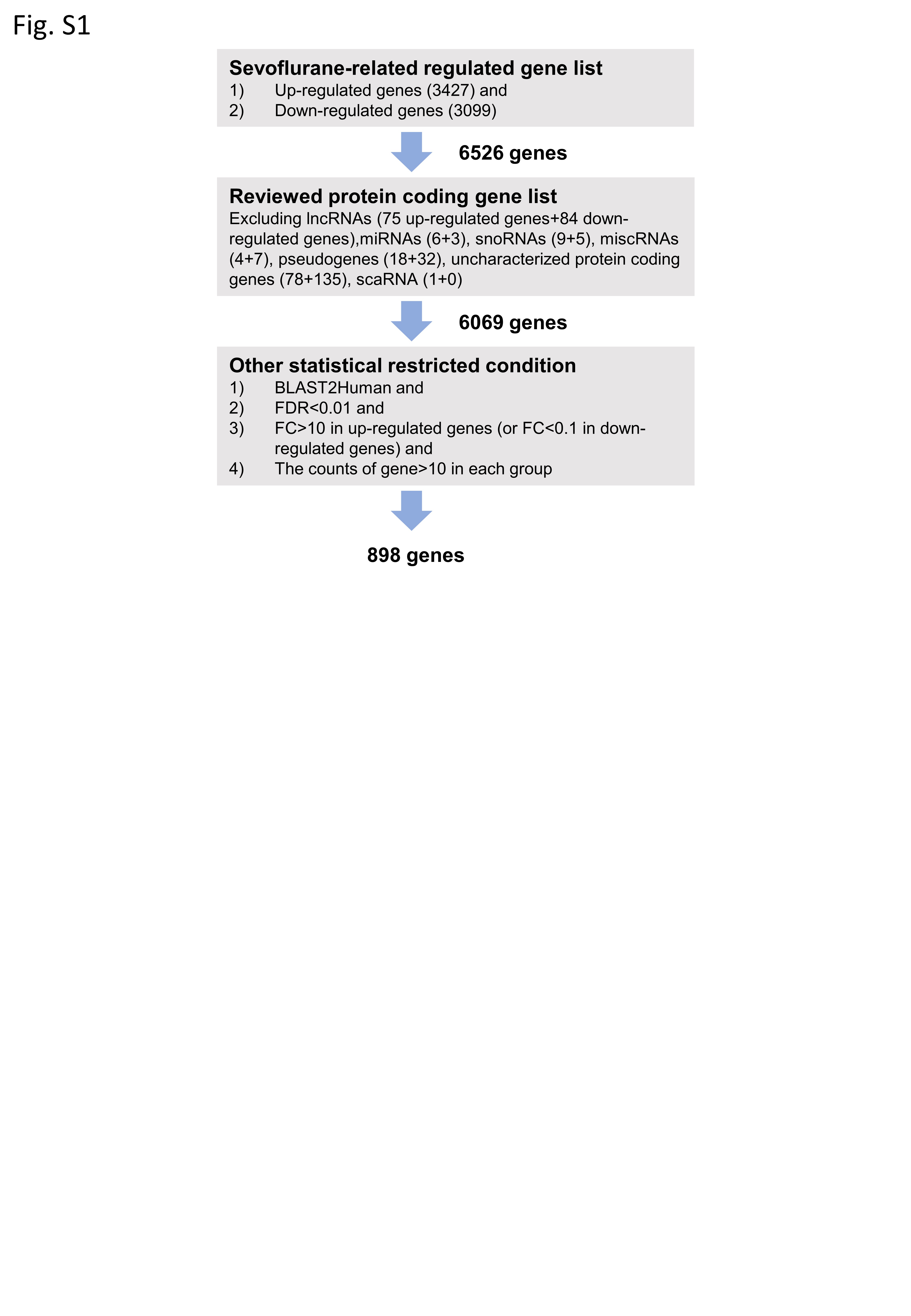
**

**Fig.S1 Working flow of selecting differentially expressed genes (DEGs) in the amygdala of macaques after sevoflurane anesthesia.**

**
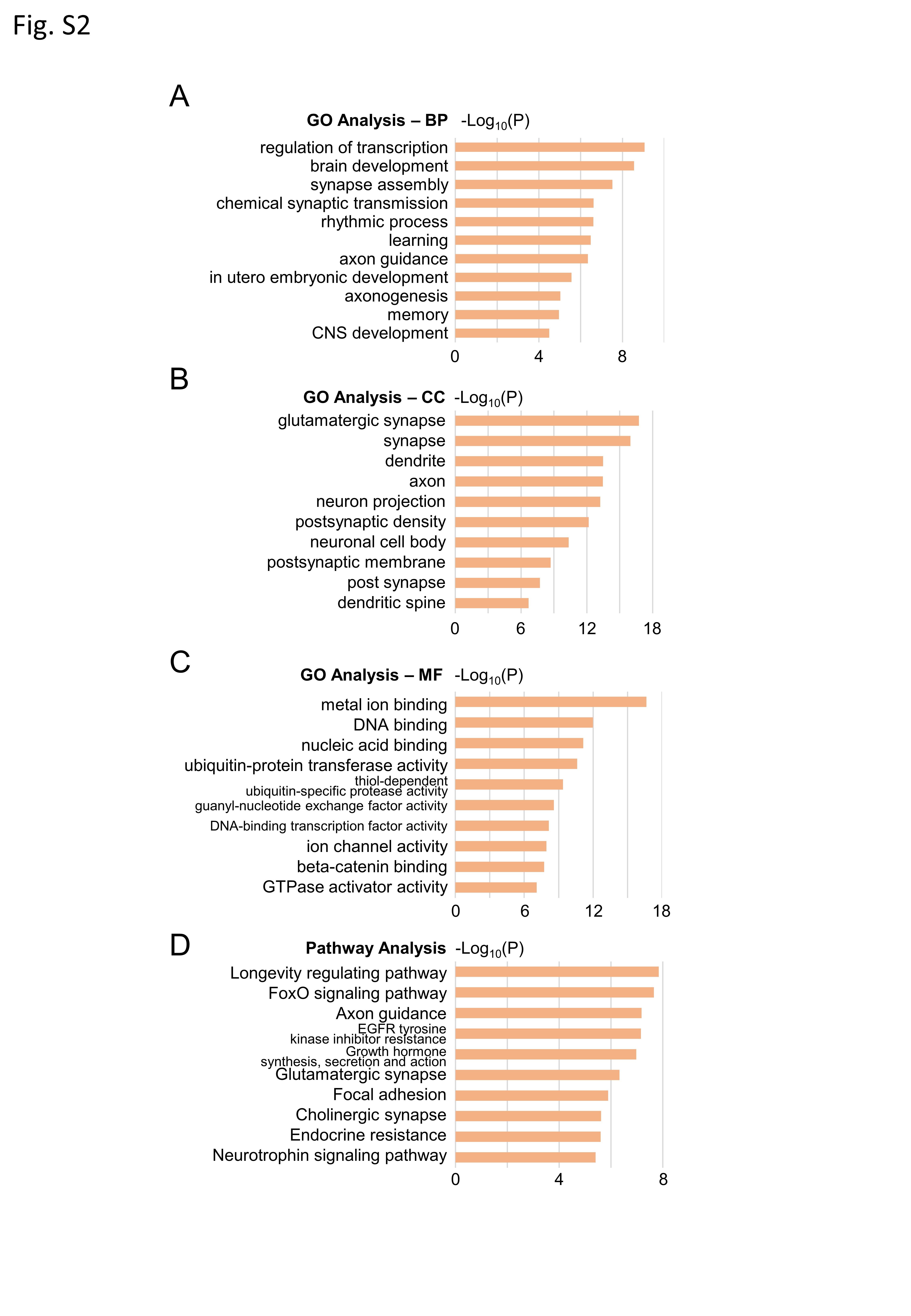
**

**Fig.S2 The Gene Ontology (GO) analysis and Kyoto encyclopedia of genes and genomes (KEGG) pathway terms of up-regulated genes in macaques’ amygdala after multiple sevoflurane anesthesia.**

(A-C) GO analysis covered three domains: biological process (BP), cellular component (CC) and molecular function (MF). Bar plot of GO enrichment in BP(A), CC(B), MF(C) in macaques’ amygdala after multiple sevoflurane anesthesia.

(D) Bar plot of KEGG pathway enrichment in macaques’ amygdala after multiple sevoflurane anesthesia.

**
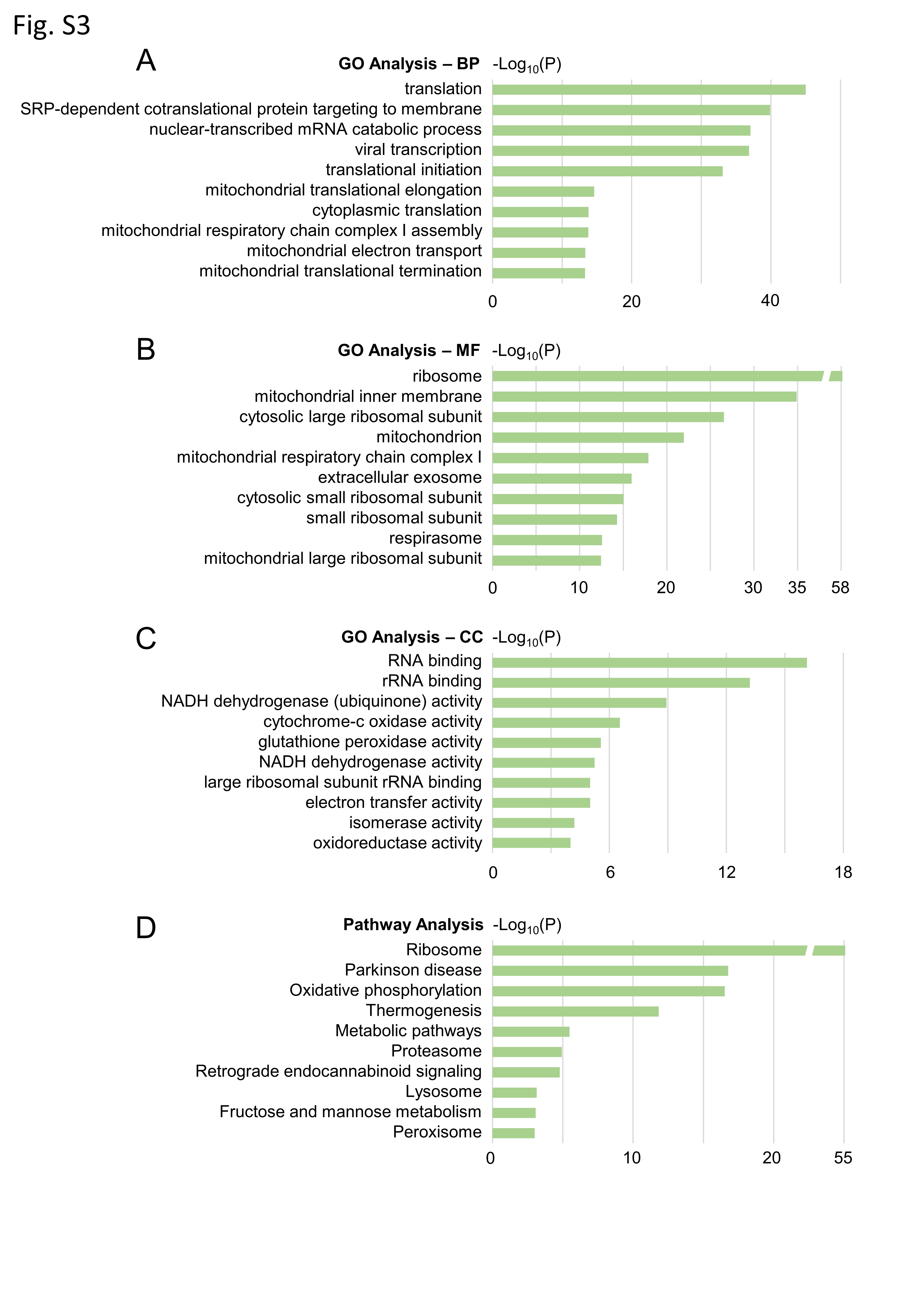
**

**Fig.S3 The GO analysis and KEGG pathway terms of down-regulated genes in macaques’ amygdala after multiple sevoflurane anesthesia.**

(A-C) Bar plot of GO enrichment in BP(A), CC(B), MF(C) in macaques’ amygdala after multiple sevoflurane anesthesia.

(D) Bar plot of KEGG pathway enrichment in macaques’ amygdala after multiple sevoflurane anesthesia.


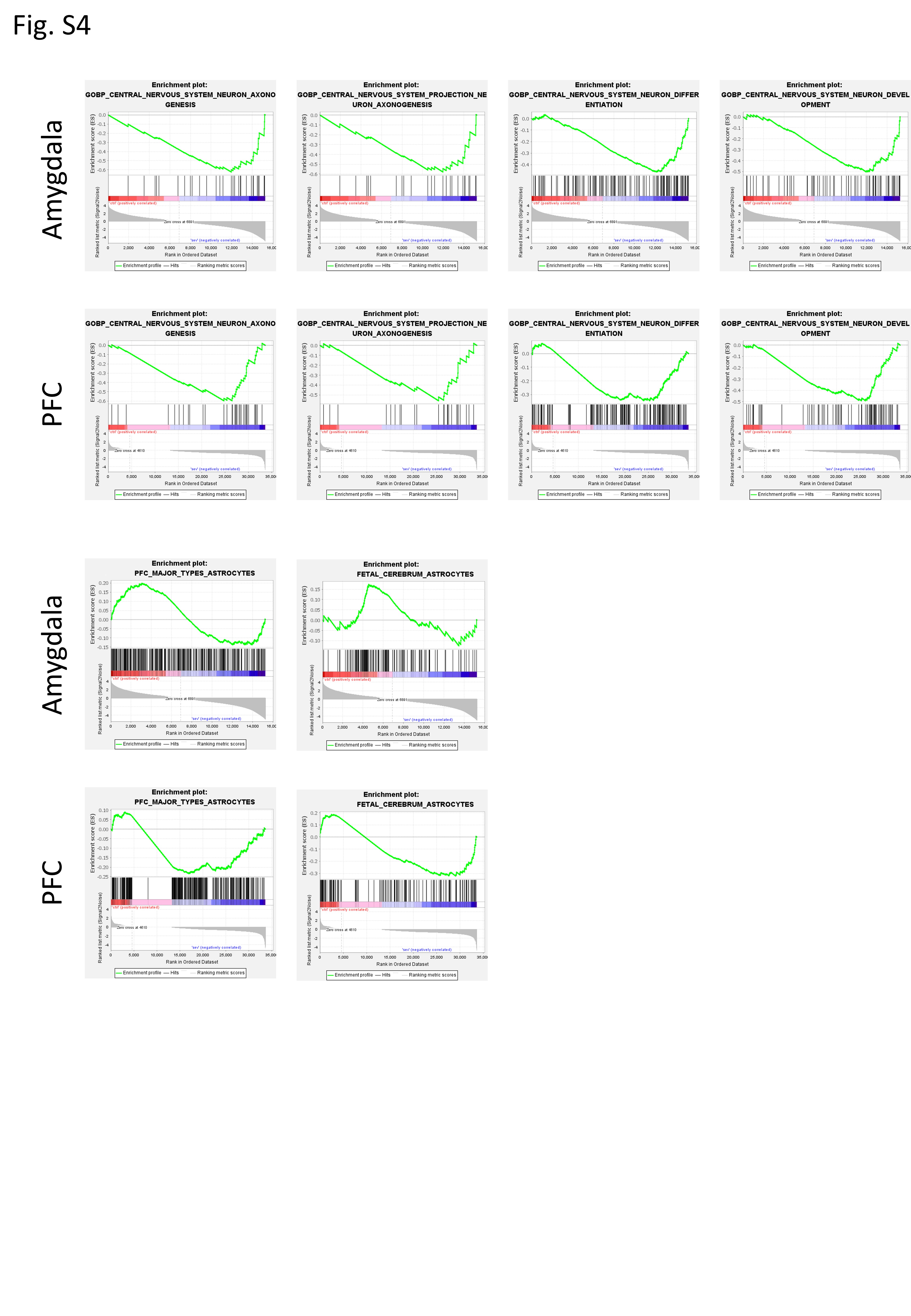


**Fig.S4 Gene set enrichment analysis (GSEA) results of DEGs enriched in amygdala and prefrontal cortex (PFC)after multiple sevoflurane anesthesia.**

**
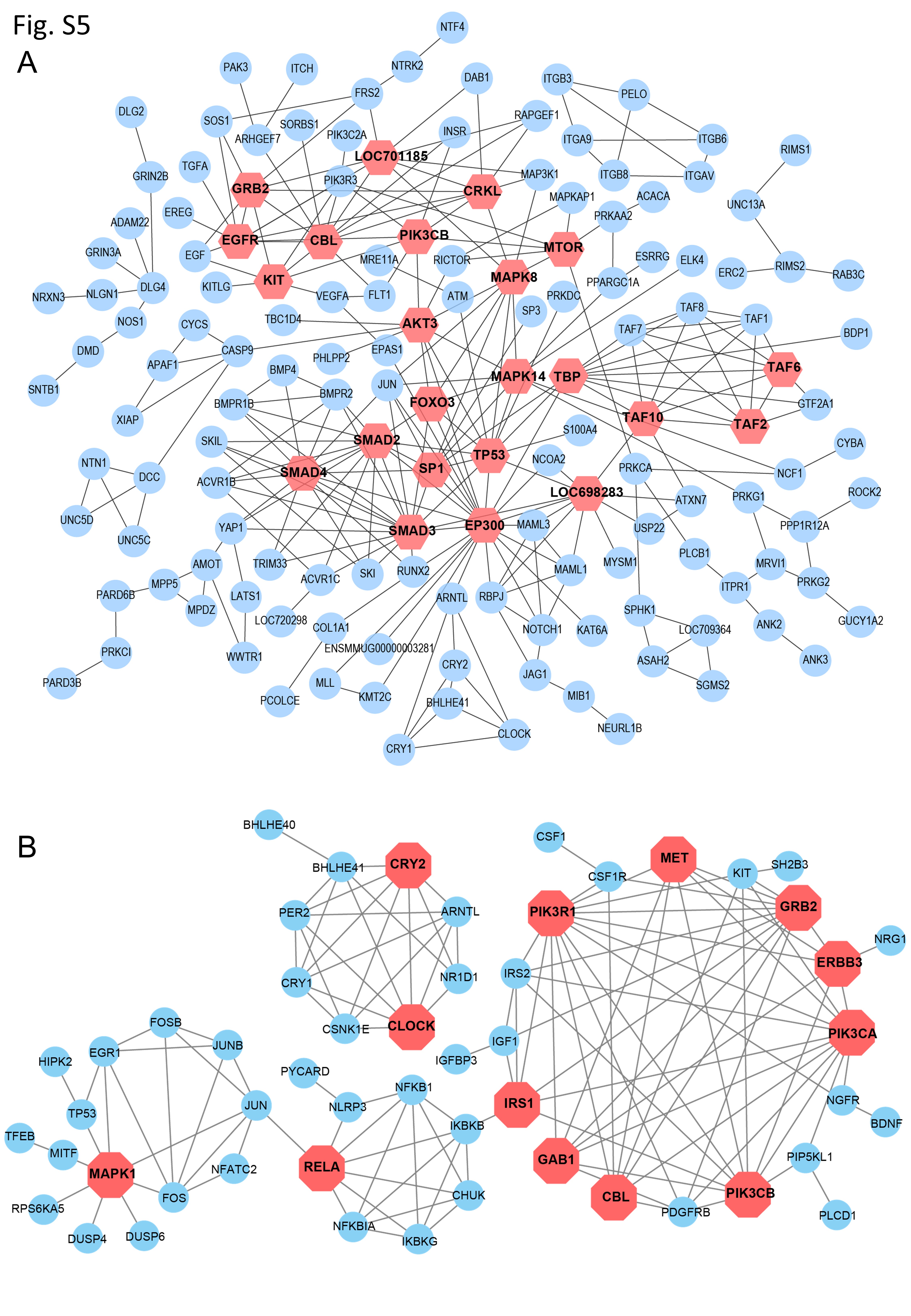
**

**Fig.S5 Protein–protein interactions (PPI) analysis of DEGs in macaques’ amygdala and PFC after multiple sevoflurane anesthesia.**

1. PPI network construction of 898 DEGs in macaques’ amygdala after multiple sevoflurane anesthesia.

(B) PPI network construction of 387 DEGs in macaques’ PFC after multiple sevoflurane anesthesia. Hub genes selected by Cytoscape are marked in red.

**
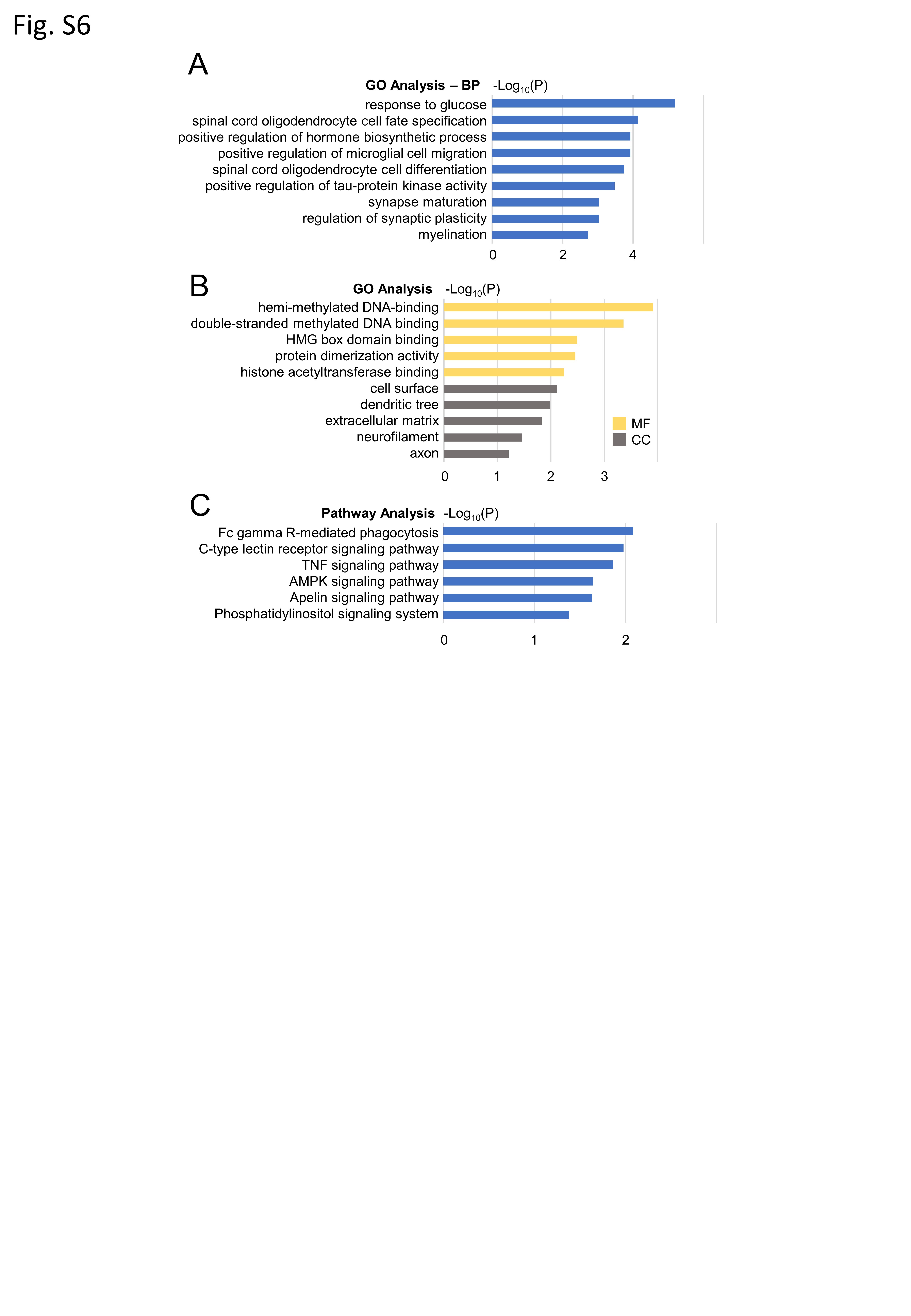
**

**Fig.S6 The GO analysis and KEGG pathway terms of the overlap DEGs in macaques’ amygdala and PFC after multiple sevoflurane anesthesia.**

(A,B) Bar plot of GO enrichment in BP(A), CC(B), MF(B) of the overlap DEGs in macaques’ amygdala and PFC after multiple sevoflurane anesthesia.

(C) Bar plot of KEGG pathway enriched terms of the overlap DEGs in macaques’ amygdala and PFC after multiple sevoflurane anesthesia.

**
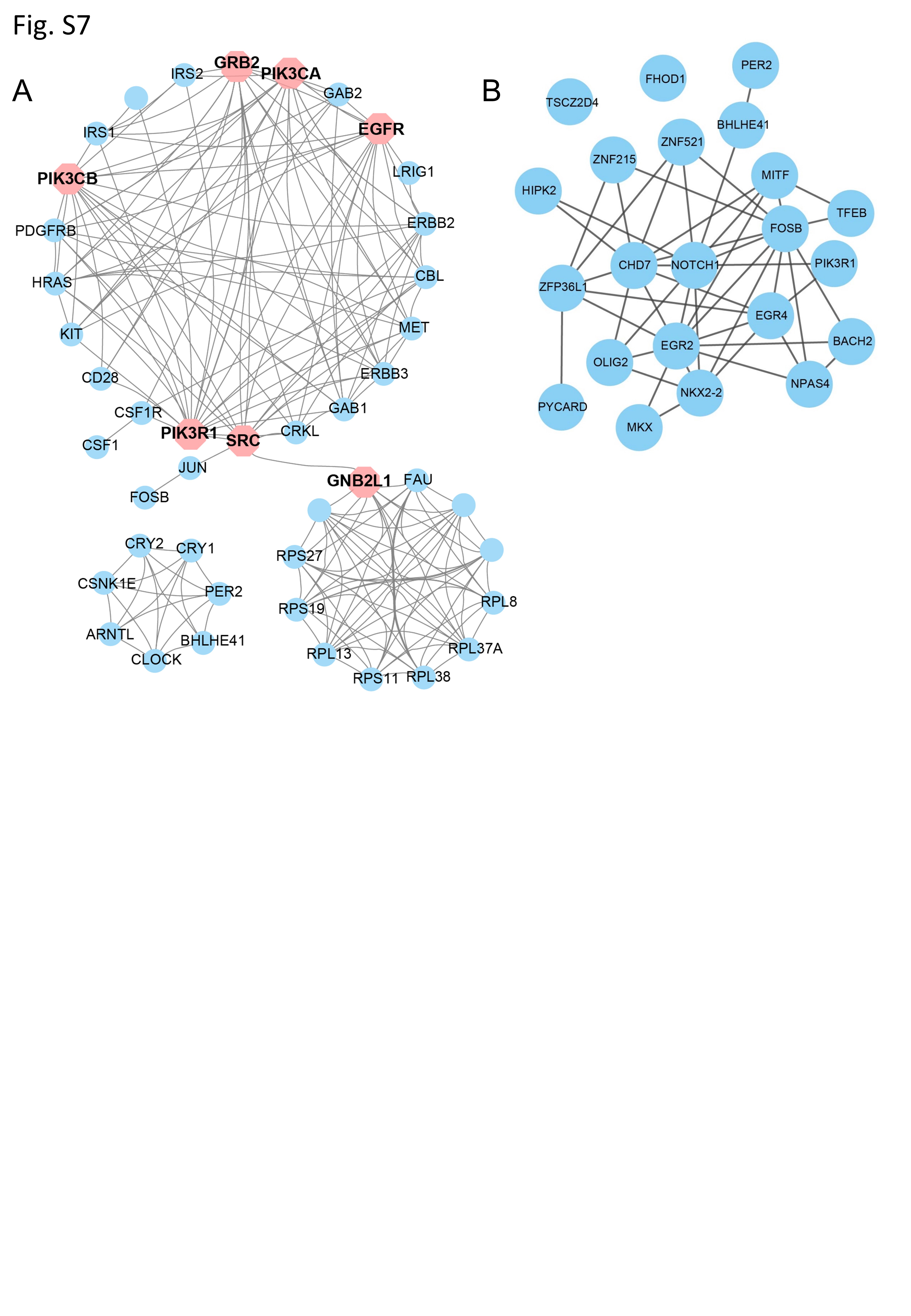
**

**Fig.S7 PPI analysis of the overlapped DEGs in macaques’ amygdala and PFC after multiple sevoflurane anesthesia.**

1. PPI network diagram of the overlapped 111 DEGs in both PFC and amygdala of macaque after multiple sevoflurane anesthesia. (B) PPI network diagram of transcription factors (TFs) in the overlap.

**
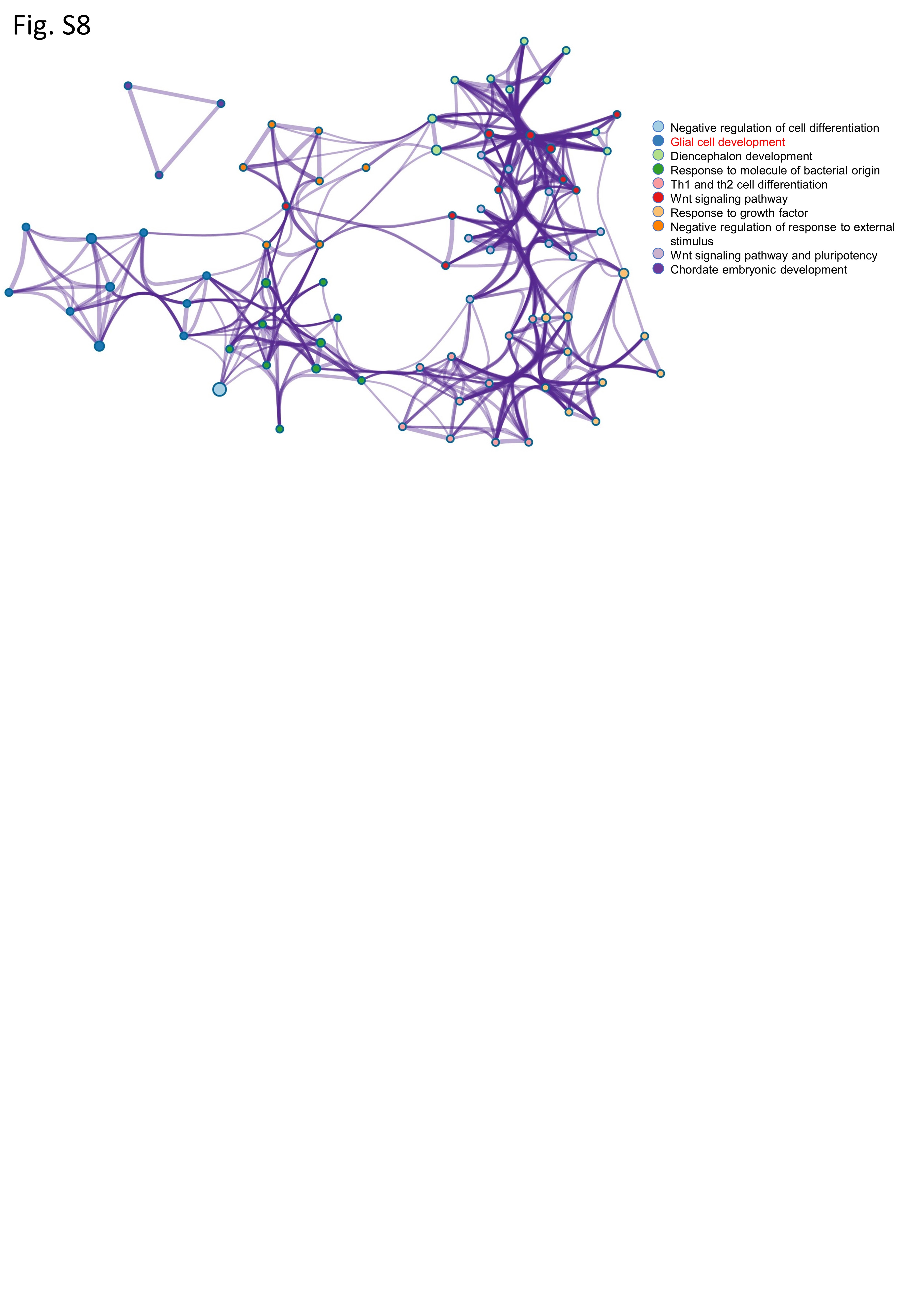
**

**Fig.S8 Functional annotation of TFs in macaques’ PFC after multiple sevoflurane anesthesia using Metascape.**

**
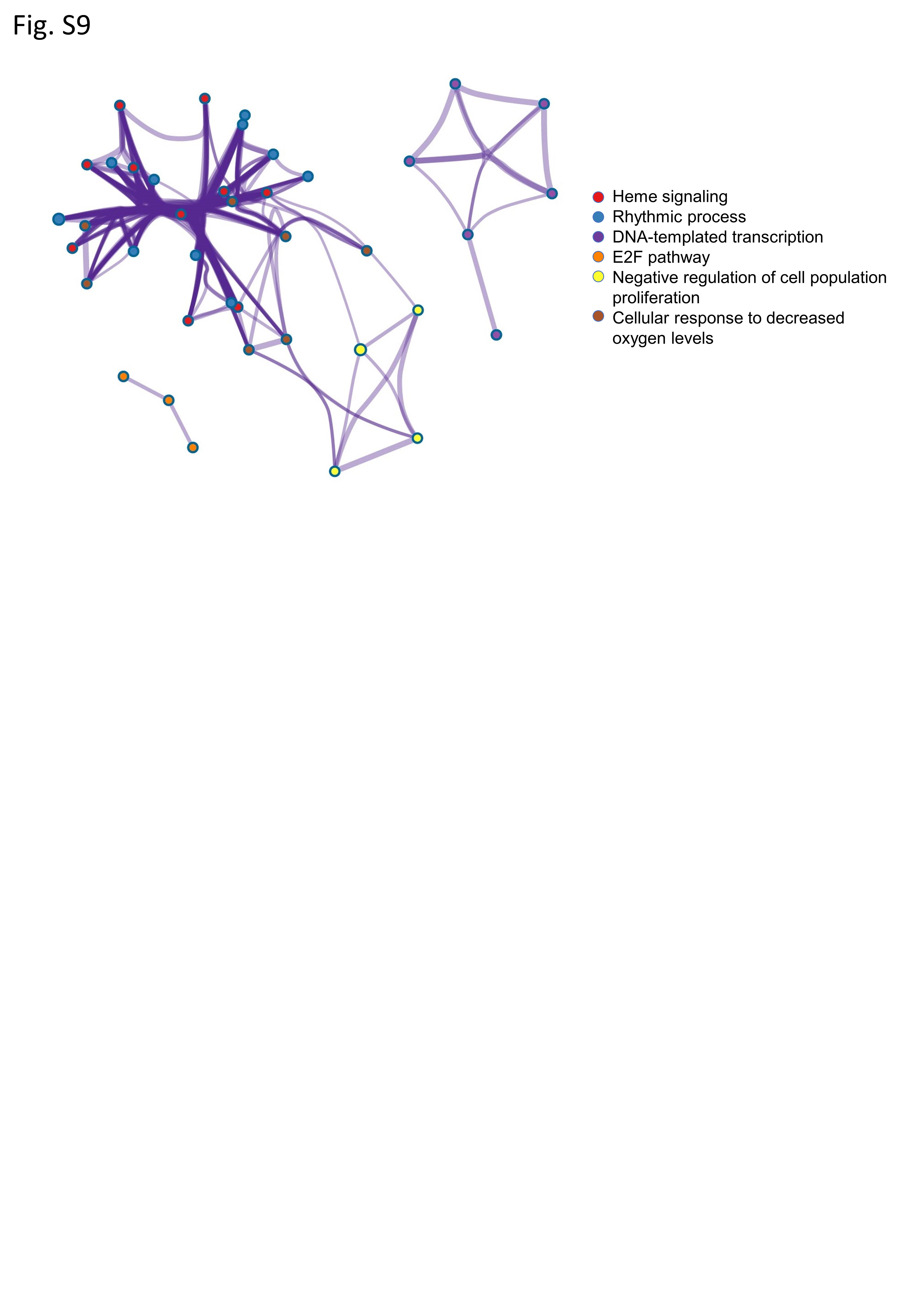
**

**Fig.S9 Functional annotation of TFs in macaques’ amygdala after multiple sevoflurane anesthesia using Metascape.**
